# Effect of the magnetic field on positron range using GATE for PET-MR

**DOI:** 10.1186/2197-7364-1-S1-A50

**Published:** 2014-07-29

**Authors:** Afroditi Eleftheriou, Charalampos Tsoumpas, Ottavia Bertolli, Εfstathios Stiliaris

**Affiliations:** Division of Medical Physics, University of Leeds, Kragujevac, UK; Department of Physics, National and Kapodistrian University of Athens, Kragujevac, Greece; Institute of Accelerating Systems & Applications (IASA), Athens, Greece

Positron range is an important spatial resolution limiting factor in PET. When imaging inside a magnetic field the positron range is non-uniformly affected. A decrease of the positron range is expected in the directions perpendicular to the direction of the magnetic field, whereas no variation is expected in the direction of the magnetic field. Monte Carlo simulations were performed to validate these expectations.

GATE simulation package [1] (version 5.0.0) was used to calculate the annihilation distribution of positrons in water, lung and rib bone. A point source placed in the centre of a spherical phantom was simulated. Six different positron emitters were used: ^11^C, ^13^ N, ^15^O, ^18^F, ^68^Ga, ^82^Rb. The simulations were performed without and with static magnetic field set in the axial direction for various field strengths. In total 105 annihilation events were simulated per configuration and the annihilation coordinates were obtained from the Geant4 output and analysed with Matlab. Previous investigations indicated an increase of the positron range along the axis of the magnetic field [2, 3]. As an attempt to confirm the previous findings, the effect of the magnetic field on the positron emission range was investigated.

GATE simulations of the positron annihilation process indicated a general reduction of the mean positron range inside the phantom volume, specifically for the high energy emitters ^82^Rb and ^68^Ga. The evaluation of the positron annihilation distance across the directions perpendicular to the magnetic field showed a reduction of the mean positron range, as theoretically expected [4, 5]. These results are compared and found in accordance with the theoretical expectations [2, 3]. Contrary to previously published results [2, 3], no increase in the positron range along the direction of the magnetic field is detected. The results of this study can be used to improve positron range correction algorithms for simultaneous PET-MR acquisition.
